# Heat Shock Protein 70 Protects the Heart from Ischemia/Reperfusion Injury through Inhibition of p38 MAPK Signaling

**DOI:** 10.1155/2020/3908641

**Published:** 2020-04-07

**Authors:** Nan Song, Jiao Ma, Xiao-wen Meng, Hong Liu, Hui Wang, Shao-yong Song, Qing-cai Chen, Hua-yue Liu, Juan Zhang, Ke Peng, Fu-hai Ji

**Affiliations:** ^1^Department of Anesthesiology, First Affiliated Hospital of Soochow University, Suzhou, Jiangsu, China; ^2^Department of Anesthesiology, Children's Hospital of Soochow University, Suzhou, Jiangsu, China; ^3^Department of Anesthesiology and Pain Medicine, University of California Davis Health System, Sacramento, CA, USA

## Abstract

**Background:**

Heat shock protein 70 (Hsp70) has been shown to exert cardioprotection. Intracellular calcium ([Ca^2+^]_i_) overload induced by p38 mitogen-activated protein kinase (p38 MAPK) activation contributes to cardiac ischemia/reperfusion (I/R) injury. However, whether Hsp70 interacts with p38 MAPK signaling is unclear. Therefore, this study investigated the regulation of p38 MAPK by Hsp70 in I/R-induced cardiac injury.

**Methods:**

Neonatal rat cardiomyocytes were subjected to oxygen-glucose deprivation for 6 h followed by 2 h reoxygenation (OGD/R), and rats underwent left anterior artery ligation for 30 min followed by 30 min of reperfusion. The p38 MAPK inhibitor (SB203580), Hsp70 inhibitor (Quercetin), and Hsp70 short hairpin RNA (shRNA) were used prior to OGD/R or I/R. Cell viability, lactate dehydrogenase (LDH) release, serum cardiac troponin I (cTnI), [Ca^2+^]_i_ levels, cell apoptosis, myocardial infarct size, mRNA level of IL-1*β* and IL-6, and protein expression of Hsp70, phosphorylated p38 MAPK (p-p38 MAPK), sarcoplasmic/endoplasmic reticulum Ca^2+^-ATPase2 (SERCA2), phosphorylated signal transducer and activator of transcription3 (p-STAT3), and cleaved caspase3 were assessed.

**Results:**

Pretreatment with a p38 MAPK inhibitor, SB203580, significantly attenuated OGD/R-induced cell injury or I/R-induced myocardial injury, as evidenced by improved cell viability and lower LDH release, resulted in lower serum cTnI and myocardial infarct size, alleviation of [Ca^2+^]_i_ overload and cell apoptosis, inhibition of IL-1*β* and IL-6, and modulation of protein expressions of p-p38 MAPK, SERCA2, p-STAT3, and cleaved-caspase3. Knockdown of Hsp70 by shRNA exacerbated OGD/R-induced cell injury, which was effectively abolished by SB203580. Moreover, inhibition of Hsp70 by quercetin enhanced I/R-induced myocardial injury, while SB203580 pretreatment reversed the harmful effects caused by quercetin.

**Conclusions:**

Inhibition of Hsp70 aggravates [Ca^2+^]_i_ overload, inflammation, and apoptosis through regulating p38 MAPK signaling during cardiac I/R injury, which may help provide novel insight into cardioprotective strategies.

## 1. Introduction

Myocardial ischemia/reperfusion (I/R) injury is a common and complicated pathophysiological phenomenon that leads to arrhythmias, myocardial stunning, and heart failure [1]. Recent studies have investigated cardioprotective therapies in the animal models of I/R-induced myocardial injury [2–4]. Despite recent progress, preventing and treating myocardial I/R injury remains an unsolved problem in the clinical settings.

Several possible underlying mechanisms include intracellular calcium ([Ca^2+^]_i_) overload, oxidative stress, inflammatory reactions, apoptosis, autophagy, and platelet aggregation and embolization [5–7]. Of these, [Ca^2+^]_i_ overload is a major etiological factor associated with Ca^2+^ homeostasis disorders, apoptosis, and other types of cell damage during cardiac I/R injury [6, 8–11]. In the heart, p38 mitogen-activated protein kinase (p38 MAPK) is involved in the regulation of cardiac contractile function. Previous studies showed that inhibition of p38 MAPK protected against myocardial I/R injury [12, 13]. Moreover, Kaikkonen et al. found that inhibition of p38 MAPK enhanced diastolic Ca^2+^ uptake and improved cardiomyocyte contractile function [14]. However, the upstream mechanism of p38 MAPK regulating [Ca^2+^]_i_ during myocardial I/R injury needs further investigations.

The 70 kDa heat shock protein (Hsp70), a member of the HSP family, is a highly conserved protein widely expressed in all living organisms. Hsp70 plays an important role in protecting cellular homeostasis from stress via its molecular chaperone functions [15]. Peng et al. showed that Hsp70 was involved in cell survival signaling during oxidative stress and I/R injury of rat cardiomyocytes [16]. Other studies found that the Hsp70 expression was upregulated in I/R-induced myocardial injury, which may help to alleviate cell apoptosis [17, 18]. In skeletal muscle regeneration, Hsp70 regulated p38 MAPK stability by interaction with MAPK-activated protein kinase 2 [19]. In the context of myocardial I/R injury, however, whether Hsp70 affects p38 MAPK signaling is unclear.

In this study, we investigated the protective role of Hsp70 and its regulation of p38 MAPK signaling during I/R-induced cardiac injury, *in vivo* and *in vitro*. We hypothesized that inhibition of Hsp70 could aggravate [Ca^2+^]_i_ overload and cell apoptosis, and these effects are medicated through the p38 MAPK signaling pathway. The results of this study may help to identify potential targets for the prevention and therapeutics of myocardial I/R injury.

## 2. Materials and Methods

### 2.1. Animals

Adult male Sprague-Dawley (SD) rats (270 ± 20 g) and neonatal rats (aged 24–48 h) were provided by the Experimental Animal Center of Soochow University (license No. SYXK Jiangsu 2017-0043) and were kept under a controlled condition (temperature of 24–26°C, relative humidity 40–60%, 12 h light–dark cycle; free access to food and water). The study protocol was approved by the Institutional Animal Care and Use Committee of Soochow University (Suzhou, Jiangsu, China). All experimental procedures were performed in accordance with the Guide for the Care of Use of Laboratory Animals (NIH publication No. 85-23, revised in 1996).

### 2.2. Isolation and Culture of Neonatal Rat Cardiomyocytes

The primary cultured cardiomyocytes were obtained as previously described [20]. Briefly, neonatal rats were anesthetized with isoflurane and euthanized by cervical dislocation. The hearts were rapidly harvested in ice-cold phosphate-buffered saline (PBS). The ventricular myocardium was minced into 1 mm^3^ pieces, followed by digestion with 0.1% collagenase type II (Sigma, St. Louis, MO, USA) at 37°C for 5 min. After digestion for 5 times, Dulbecco's modified Eagle's medium (DMEM, HyClone, USA) containing 10% fetal bovine serum (FBS, Biological Industries, Israel) was added. The cell suspension was centrifuged at 500 g at 4°C for 10 min and resuspended in medium containing 10% FBS. After incubation at 37°C for 120 min, fibroblasts were removed and nonadherent cells were collected. To inhibit fibroblast growth, 0.1 mM 5-bromodeoxyuridine (5-BrdU, Sigma, St. Louis, MO, USA) was added. Cardiomyocytes were incubated at 37°C in 95% air and 5% CO_2_ for subsequent experiments.

### 2.3. Oxygen-Glucose Deprivation/Reoxygenation (OGD/R)

To induce OGD/R, the cardiomyocytes were treated with glucose-free DMEM (Gibco, Carlsbad, CA, USA) and incubated at 37°C in a hypoxia chamber containing 95% N_2_ and 5% CO_2_, as previous described [21]. Cells were subjected to OGD for 6 h, followed by reoxygenation in normal DMEM medium for 2, 12, or 24 h, respectively.

### 2.4. Myocardial I/R

Rats were anesthetized with 50 mg/kg sodium pentobarbital intraperitoneally. Then, the rats were intubated and the lungs were ventilated with oxygen-enriched room air (21% O_2_, tidal volume = 20 ml/kg, ratio = 1 : 1, and frequency = 70 breaths/min) on a small animal ventilator (R407, RWD, China). After a left thoracotomy, the left anterior artery was ligated by using a 5-0 Prolene suture to induce left ventricular myocardial ischemia for 30 min. Subsequently, the ligature was removed to restore the blood flow for 30 min, 2 h, or 24 h, respectively. The sham rats underwent the same surgical procedure without ligation. Throughout the surgery, rat body temperature was maintained at 37°C by using a heating pad (Physitemp Instruments, USA). Local infiltration with 1% lidocaine 3 ml was used for postoperative analgesia.

### 2.5. Experimental Protocol

**Figure 1:**
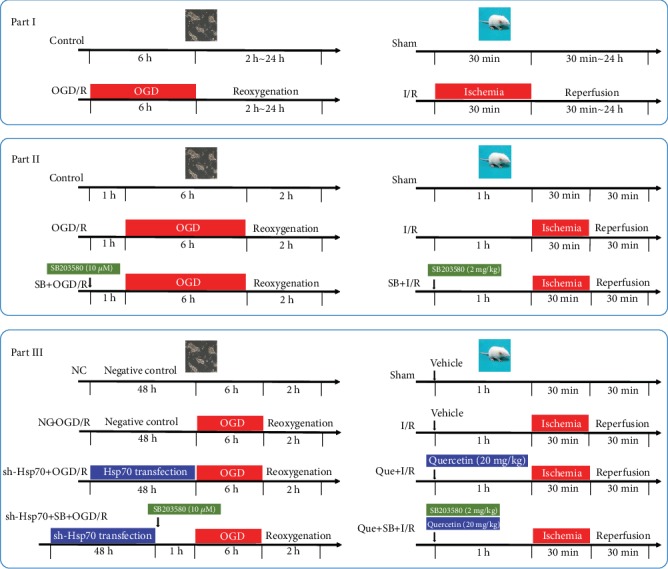
Study protocol. Part I: neonatal rat cardiomyocytes were subjected to OGD/R, and rats underwent myocardial I/R. Part II: the p38 MAPK inhibitor (SB203580) was used 1 h prior to OGD in cells or before myocardial ischemia in rats. Part III: cells were transfected with Hsp70 shRNA for 48 h and treated with SB203580 1 h prior to OGD, and rats received Quercetin (an Hsp70 inhibitor) and SB203580 1 h prior to myocardial ischemia. OGD/R: oxygen-glucose deprivation/reoxygenation; I/R: ischemia/reperfusion.

The protocol of this study is shown in Figure 1, including *in vitro* and *in vivo* experiments in three parts. A computer-generated randomization table was used to assign the cells or rats into different groups. The following parameters were assessed: cell viability, lactate dehydrogenase (LDH) release, myocardial infarct size, serum cardiac troponin I (cTnI), level of [Ca^2+^]_i_, mRNA level of interleukin 1*β* (IL-1*β*) and interleukin-6 (IL-6), apoptosis rate, and protein expression of Hsp70, p-p38 MAPK, p38 MAPK, sarcoplasmic/endoplasmic reticulum Ca^2+^-ATPase (SERCA2), signal transducer and activator of transcription3 (STAT3), p-STAT3, cleaved caspase3, and caspase3. In each part, separate experiments were performed for the sham and I/R groups in rats and control and OGD/R groups in cells.

Part 1: Neonatal rat cardiomyocytes were randomly divided into a control group and four OGD/R groups. The control group received normal culture. In the OGD/R groups, cells underwent OGD for 6 h followed by reoxygenation for 0, 2, 12, and 24 h, respectively. Rats were randomly divided into a sham group and three I/R groups. In the I/R groups, rats were subjected to myocardial ischemia for 30 min followed by reperfusion for 30 min, 2 h, or 24 h, respectively.

Part 2: To investigate the role of p38 MAPK in cardiac I/R injury, the effects of SB203580, a p38 MAPK inhibitor, on OGD/R-induced cell injury and I/R-induced myocardial injury were evaluated [22–24]. Cells were randomly divided into three groups: control group, OGD/R group, and SB203580 + OGD/R group (incubation with 10 *μ*M SB203580 1 h prior to OGD). Rats were randomly divided into three groups: sham group, I/R group, and SB203580 + I/R group (intraperitoneal injection of 2 mg/kg SB203580 1 h prior to myocardial ischemia).

Part 3: To explore the role of Hsp70 and its relationship with p38 MAPK in cardiac I/R injury, the effects of Hsp70 knockdown or inhibition, in combination with SB203580, on OGD/R-induced cell injury or I/R-induced myocardial injury were evaluated.

or Hsp70 knockdown in cells, the sequence of short hairpin RNA (shRNA, Genechem, Shanghai, China) targeting Hsp70 (NM_031971) was 5′GCCCAAGGTGCAGGTGAACTA-3′. Cells were randomly divided into four groups: negative control group (negative control transfection for 48 h followed by culture in normal conditions), NC + OGD/R group (negative control transfection for 48 h prior to OGD/R), sh − Hsp70 + OGD/R group (Hsp70-shRNA transfection for 48 h prior to OGD/R), and sh − Hsp70 + SB + OGD/R group (Hsp70 shRNA transfection for 48 h prior to 10 *μ*M SB2203580 treatment, followed by OGD/R). Transfection was performed by using the jetOPTIMUS® transfection regent (Polyplus-transfection®SA, Strasbourg, Bas-Rhin, France).

For Hsp70 inhibition in rats, 20 mg/kg quercetin (Apexbio, Houston, TX, USA) was intraperitoneally injected [25, 26]. Rats were randomly divided into four groups: sham group (sham operation with normal saline injection), I/R group (myocardial I/R with saline injection), Quercetin + I/R group (Quercetin injection 1 h prior to myocardial I/R), and Quercetin + SB203580 + I/R group (Quercetin and SB203580 injection 1 h prior to myocardial I/R).

### 2.6. Cell Viability

Cell viability was assessed by using the Cell Counting Kit (CCK)-8 (Dojindo Laboratories, Kumamoto, Japan) according to the manufacturer's instructions. Briefly, cells were cultured in 96-well plates. At the end of reoxygenation, 10 *μ*L CCK8 solution was added to each well, followed by 1 h of incubation at 37°C. The absorbance values were measured at 450 nm by using a microplate reader (Molecular Devices, Sunnyvale, CA, USA).

### 2.7. LDH Release

At the end of reoxygenation, LDH release were detected by using the LDH Assay kit (ab65393, Abcam, Cambridge, UK). The cell culture plates were centrifuged at 600 g for 10 min, and supernatants (10 *μ*l/well) were extracted into another 96-well plate. Then, 100 *μ*l LDH reaction mix was added to each well and incubated at room temperature for 30 min. The absorbance values were measured at 490 nm on the microplate reader.

### 2.8. Blood Analysis

At the end of reperfusion, 0.5 ml blood sample was taken from the abdominal aorta and analyzed by using a blood gas analyzer (Radiometer, ABL80 FLEX, Carlsbad, CA, USA). The values of pH, partial pressure of arterial oxygen (PO_2_), partial pressure of arterial carbon dioxide (PaCO_2_), hematocrit (Hct), hemoglobin [6], sodium (Na^+^), and potassium (K^+^) were measured.

### 2.9. Myocardial Infarct Size

Myocardial infarct size was determined by using the Evans blue/2,3,5-triphenyl tetrazolium chloride (TTC) staining, as previously described [27]. At the end of reperfusion, the LAD was reoccluded and 3 ml Evans blue dye (2% w/v, E2129, Sigma-Aldrich Corp., St. Louis, MO, USA) was injected via the inferior vena cava to identify the area at risk (AAR, area not stained by Evans blue). The heart was rapidly removed and frozen at -20°C for 30 min. After atrium removal, the left ventricle was sectioned into slices of 2 mm thick. The slices were immediately incubated with phosphate-buffered 1% TTC (T8877, Sigma-Aldrich Corp., St. Louis, MO, USA) at 37°C for 30 min, fixed in 10% formalin solution overnight, and then photographed. The infarct size was calculated as the ratio of infarct area (IA, area not stained by TTC) to AAR. The images were analyzed by using the Image J software (version 1.48, National Institutes of Health, Bethesda, MD, USA).

### 2.10. Western Blotting

Total protein was extracted by using the RIPA reagents (P0013B, Beyotime, Shanghai, China), and the protein concentration was determined with a bicinchoninic acid reagent kit (P1002, Beyotime, Shanghai, China) [2]. Proteins were then separated by sodium dodecyl sulphate-polyacrylamide gel electrophoresis on 10% gels and transferred to polyvinylidene fluoride membranes (Millipore Corp., Bedford, MA) at 200 mA for 80 min at 4°C. After blocking with 5% milk for 2 h at room temperature, the bands were incubated overnight at 4°C with the following specific primary antibodies: Hsp70 (1 : 1000, ab2787, Abcam, Cambridge, MA, USA), p38 MAPK (1 : 1000, ab32142, Abcam, Cambridge, MA, USA), p-p38 MAPK (1 : 1000, 4511, Cell Signaling Technology, Beverly, MA, USA), SERCA2a (1 : 500, ab2861, Abcam, Cambridge, MA, USA), STAT3 (1 : 1000, ab68153, Abcam, Cambridge, MA, USA), pSTAT3 (1 : 1000, 9145, Cell Signaling Technology, Beverly, MA, USA), and *β*-tubulin (1 : 1000, 2148, Cell Signaling Technology, Beverly, MA, USA). The membranes were then incubated with horseradish peroxidase-conjugated secondary antibodies (1 : 5000, Santa Cruz Biotechnology, CA, USA) for 2 h at room temperature. Finally, the bands were visualized by using the ChemiDocTM XRS+ System (Bio-Rad, Richmond, CA) with an enhanced chemiluminescence kit (Beyotime, Shanghai, China). The densities of protein bands were normalized to *β*-tubulin as control.

### 2.11. Quantitative Real-Time Polymerase Chain Reaction

Total RNA was extracted by using the TRIZOL reagent (Invitrogen; Thermo Fisher Scientific, Inc. Waltham, MA, USA), as previously described [20]. RNA quantification and purity control were determined by absorbance at 260 and 280 nm. Reverse transcription was performed by using a cDNA Synthesis Kit (Applied Biological Materials, Richmond, BC, Canada). RNA samples were added to 5× All-in-one and DEPC water in a 20 *μ*l reaction system for reverse transcription to cDNA. Quantitative real-time polymerase chain reaction (PCR) was conducted with EvaGreen qPCR MasterMix (Applied Biological Materials, Richmond, BC, Canada) in a 10 *μ*l reaction volume on the Roche Light Cycler R480 System (Roche, Bedford, MA, USA). The amplification conditions were predenaturation at 95°C for 10 s, denaturation at 58°C for 15 s, and annealing at 75°C for 20 s for 40 cycles. The expression of target gene was analyzed by using the 2^−*Δ*ΔCT^ method and normalized to *β*-tubulin. Three replicates were tested for each sample. The primers were provided by Shanghai Shenggong Co., Ltd., and the sequences were: IL-1*β*, 5'-ATCTCACAGCAGCATCTCGACAAG-3'(forward), and 5'-CACACTAGCAGGTCGTCATCATCC-3'(reverse); IL-6, 5'-AGGAGTGGCTAAGGACCAAGACC-3'(forward), and 5'-TGCCAGGTAGACCTCATAGTGACC-3'(reverse); and *β*-tubulin, 5'-GGGAGGTGATAAGCGATGAA-3'(forward), and 5'-AGGGACATACTTGCCACCTG-3'(reverse).

### 2.12. TUNEL Assay

At the end of reperfusion or reoxygenation, the cardiac tissues or cardiomyocytes were fixed in 4% formaldehyde at room temperature for 24 h, embedded in paraffin, and cut into 4 *μ*m slices. Myocardial apoptosis was assessed by terminal deoxynucleotidyl transferase-mediated dUTP nick-end labeling (TUNEL) assay with the *in situ* Cell Death Detection kit (Roche Diagnostics, Basel, Switzerland), as previously described [28]. The nuclei were dyed with 4′,6-Diamidino-2-Phenylindole (DAPI, Beyotime, Shanghai, China). Samples were photographed under a fluorescence microscope (Nikon, Tokyo, Japan). Cell nuclei were counted in four random and nonoverlapping fields. The ratio of TUNEL-positive cells to total number of cells was calculated as the apoptosis index.

### 2.13. Intracellular Calcium Assay

The level of [Ca^2+^]_i_ in cardiomyocytes was measured by using the flow cytometry method [29]. Briefly, cells were collected and washed with Hank's balanced salt solution (HBSS) three times. Then, cells were incubated with 5 *μ*M Fluo-3/AM (Sigma, St. Louis, MO, USA) solution at 37°C for 30 min. After incubation, the cells were resuspended in HBSS buffer and analyzed by using a flow cytometry (BD Biosciences San Jose, CA, USA). The fluorescence was excited at 488 nm and examined at 525 nm.

To analyze the [Ca^2+^]_i_ level in rat left ventricular tissues, the number of free calcium-cresolphthalein complexone formed within the tissues were detected by using the calcium detection assay kit (ab102505, Abcam, Cambridge, MA, USA), according to the manufacturer's instructions. Briefly, left ventricular samples were harvested at the end of reperfusion. After treatment with calcium detection solution 1 ml for 3 min, the samples were centrifuged at 12000 g at 4°C for 3 min, and the supernatant was collected. Supernatant 50 *μ*l, chromogenic reagent 90 *μ*l, and buffer solution 60 *μ*l were cultured in 96-well plates at room temperature for 10 min. The absorbance values were measured at 575 nm on the microplate reader.

### 2.14. Enzyme-Linked Immunosorbent Assay

Serum samples were collected and frozen at -80°C before assays. A commercial ELISA kit (Life diagnostics, Lincoln, USA) was used as previously described [30]. The absorbance values were measured at 450 nm on a microplate reader. The cTnI concentrations were determined by using a standard curve.

### 2.15. Statistical Analysis

All data were expressed as mean ± standard error of the mean (SEM) and analyzed by using the GraphPad Prism software (version 7.0, GraphPad, San Diego, CA, USA). Data were compared by using one-way or two-way analysis of variance followed by Bonferroni or Dunnet posttest, as appropriate. A two-tailed value of *P* < 0.05 was considered statistically significant.

## 3. Results

### 3.1. Upregulated Hsp70 Protein Expression and p38 MAPK Phosphorylation during OGD/R-Induced Injury in Cardiomyocytes

**Figure 2:**
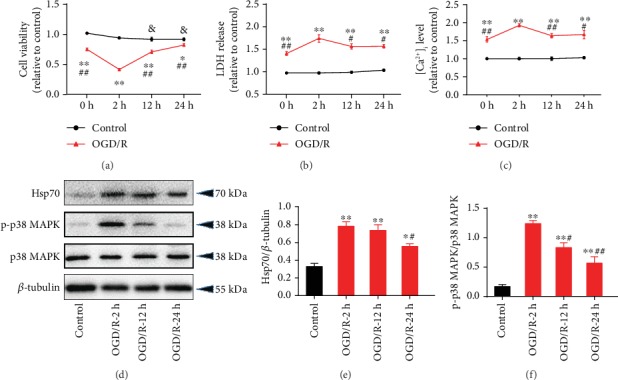
OGD/R-induced cell injury and increased protein expression of Hsp70 and phosphorylated p38 MAPK. (a) Decreased cell viability following OGD/R (*n* = 8). (b) Increased LDH release following OGD/R (*n* = 8). (c) Elevated [Ca^2+^]_i_ level following OGD/R (*n* = 5). (d) Representative western blot bands. (e) Upregulated Hsp70 protein expression following OGD/R (*n* = 5). (f) Upregulated p-p38 MAPK protein expression following OGD/R (*n* = 5). Data are shown as means ± SEM. ^∗^*P* < 0.05, ^∗∗^*P* < 0.01 vs. the control group; ^#^*P* < 0.05, ^##^*P* < 0.01 vs. the OGD/R-2 h group; ^&^*P* < 0.05 vs. the control group at 0 h. OGD/R: oxygen-glucose deprivation/reoxygenation.

In neonatal rat cardiomyocytes, OGD/R treatment significantly resulted in lower cell viability (Figure 2(a)) and resulted in higher LDH release (Figure 2(b)) and level of [Ca^2+^]_i_ (Figure 2(c)). Of the three OGD/R groups, OGR/R-2h led to the lowest cell viability and highest LDH release and [Ca^2+^]_i_ level. Besides, cells subjected to OGD/R showed significantly increased protein expression of Hsp70 and p-p38 MAPK, with the highest protein expression in the OGD/R-2h group (Figures 2(d)–2(f)). Based on these findings, OGD for 6 h followed by reoxygenation for 2 h was selected for the subsequent cell experiments.

### 3.2. Increased Protein Expression of Hsp70 and Phosphorylated p38 MAPK during I/R-Induced Myocardial Injury in Rats

**Figure 3:**
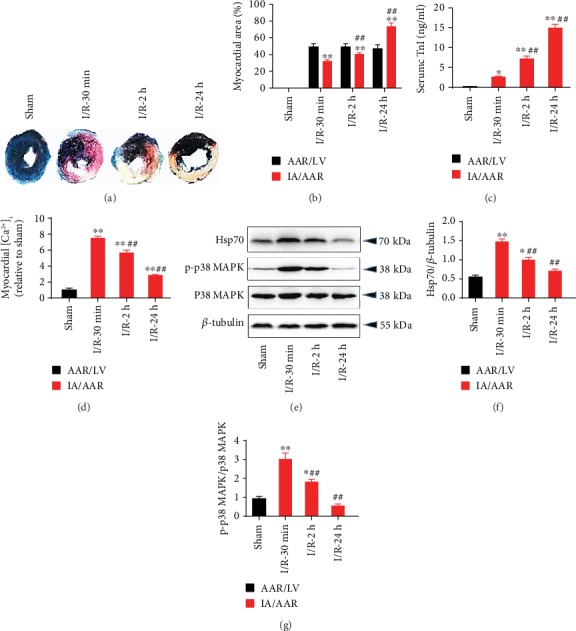
I/R-induced rat myocardial injury and upregulation of Hsp70 protein and p38 MAPK phosphorylation. (a) Representative Evans blue/TTC staining images. (b) Elevated myocardial infarct size following myocardial I/R. (c) Increased serum cTnI following myocardial I/R. (d) Increased [Ca^2+^]_i_ level following myocardial I/R. (e) Representative western blot bands. (f) Upregulated Hsp70 protein expression following myocardial I/R. (g) Upregulated p-p38 MAPK protein expression following myocardial I/R. *n* = 5. Data are shown as means ± SEM. ^∗^*P* < 0.05, ^∗∗^*P* < 0.01 vs. the sham group; ^#^*P* < 0.05, ^##^*P* < 0.01 vs. the I/R-30 min group. I/R: ischemia/reperfusion; AAR: area at risk; LV: left ventricle; IA: infarct area.

As shown in Table 1, oxygenation and homeostasis were well maintained in all groups during the study period. Myocardial I/R resulted in significant myocardial infarct size (IA/AAR) at 30 min, 2 h, and 24 h of reperfusion, although AAR/left ventricle (LV) were comparable among the I/R groups (Figures 3(a)–3(b)). Compared to the sham rats, the myocardial I/R rats showed significantly higher levels of serum cTnI (Figure 3(c)) and [Ca^2+^]_i_ in the myocardium (Figure 3(d)). During the reperfusion process, serum cTnI level peaked at 24 h of reperfusion while myocardial [Ca^2+^]_i_ level decreased over time. Moreover, the protein expression of Hsp70 and p-p38 MAPK were significantly increased at 30 min and 2 h of reperfusion and returned to normal value at 24 h, with the most obvious level at 30 min (Figures 3(e)–3(g)). Based on these findings, ischemia for 30 min followed by reperfusion for 30 min was selected for the following experiments in rats.

### 3.3. Inhibition of p38 MAPK Alleviated OGD/R-Induced Cell Injury, [Ca^2+^]_i_ Overload, and Apoptosis in Cardiomyocytes

**Figure 4:**
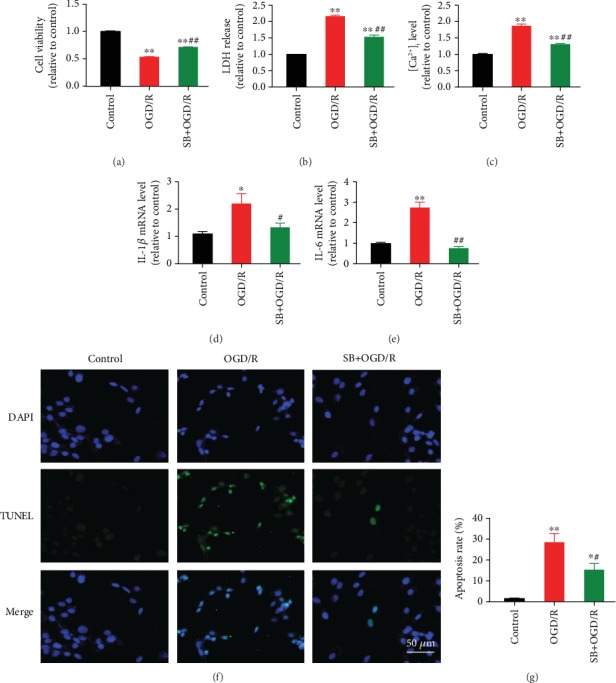
p38 MAPK inhibition alleviated OGD/R-induced injury, [Ca^2+^]_i_ overload, and apoptosis in cardiomyocytes. (a) SB203580, a p38 MAPK inhibitor, increased cell viability during OGD/R (*n* = 8). (b) SB203580 reduced OGD/R-induced LDH release (*n* = 8). (c) SB203580 inhibited OGD/R-induced [Ca^2+^]_i_ elevation (*n* = 5). (d and e) SB203580 reduced IL-1*β* and IL-6 mRNA expression during OGD/R (*n* = 5). (f) Representative TUNEL staining images. Scale bar = 50 *μ*m. (g) SB203580 inhibited OGD/R-induced cell apoptosis (*n* = 5). Data are shown as means ± SEM. ^∗^*P* < 0.05, ^∗∗^*P* < 0.01 vs. the control group; ^#^*P* < 0.05, ^##^*P* < 0.01 vs. the OGD/R group. OGD/R: oxygen-glucose deprivation/reoxygenation.

**Figure 5:**
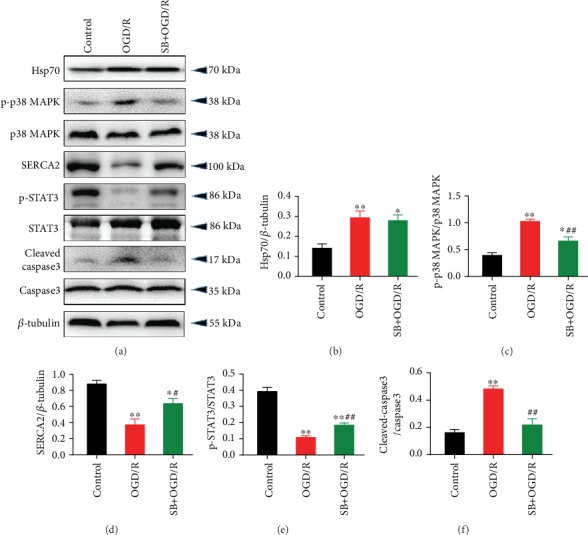
p38 MAPK inhibition reversed OGD/R-induced protein expression of p-p38 MAPK, SERCA2, p-STAT3, and cleaved caspase3, but not Hsp70, in cardiomyocytes. (a) Representative western blot bands. (b) Hsp70 protein expression. (c) p-p38 MAPK protein expression. (d) SERCA2 protein expression. (e) p-STAT3 protein expression. (f) Cleaved caspase3 protein expression. *n* = 5. Data are shown as means ± SEM. ^∗^*P* < 0.05, ^∗∗^*P* < 0.01 vs. the control group; ^#^*P* < 0.05, ^##^*P* < 0.01 vs. the OGD/R group. OGD/R: oxygen-glucose deprivation/reoxygenation.

Pretreatment with SB203580, a p38 MAPK inhibitor, significantly increased cell viability (Figure 4(a)) and reduced LDH release (Figure 4(b)) and [Ca^2+^]_i_ overload (Figure 4(c)) during OGD/R-induced injury in neonatal rat cardiomyocytes. Also, OGD/R led to activation of IL-1*β* (Figure 4(d)) and IL-6 (Figure 4(e)) as well as significant cell apoptosis (Figures 4(f)–4(g)), which was partly blocked by SB203580. Cells subjected to OGD/R exhibited increased protein expression of Hsp70, p-p38 MAPK, and cleaved-caspase3 and decreased expression of SERCA2 and p-STAT3, while pretreatment with SB203580 reversed the changes in these proteins except for Hsp70 (Figure 5).

### 3.4. Inhibition of p38 MAPK Attenuated I/R-Induced Myocardial Injury, [Ca^2+^]_i_ Overload, and Apoptosis in Rats

**Figure 6:**
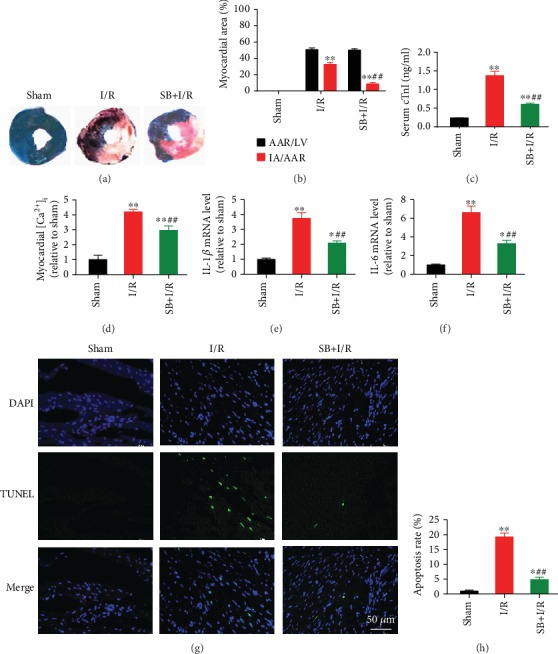
p38 MAPK inhibition attenuated I/R-induced myocardial injury, [Ca^2+^]_i_ overload, and apoptosis in rat hearts. (a) Representative Evans blue/TTC staining images. (b) SB203580 reduced I/R-induced myocardial infarct size. (c) SB203580 reduced serum cTnI level during myocardial I/R. (d) SB203580 reduced [Ca^2+^]_i_ level during myocardial I/R. (e and f) SB203580 inhibited myocardial I/R-induced IL-1*β* and IL-6 mRNA expression. (g) Representative TUNEL staining images. Scale bar = 50 *μ*m. (h) SB203580 inhibited I/R-induced myocardial apoptosis. *n* = 5. Data are shown as means ± SEM. ^∗^*P* < 0.05, ^∗∗^*P* < 0.01 vs. the sham group; ^##^*P* < 0.01 vs. the I/R group. I/R: ischemia/reperfusion; AAR: area at risk; LV: left ventricle; IA: infarct area.

**Figure 7:**
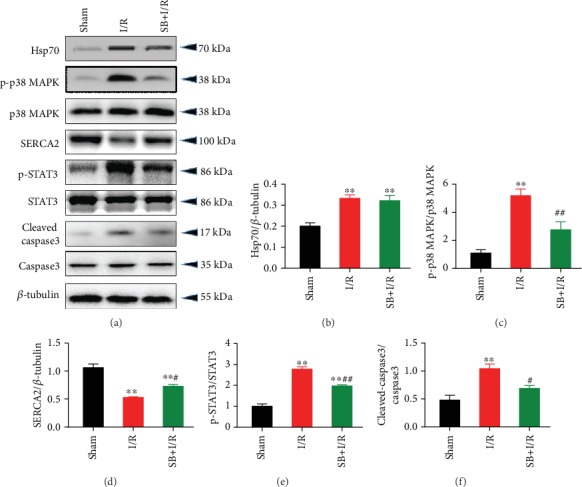
p38 MAPK inhibition reversed myocardial I/R-induced protein expression of p-p38 MAPK, SERCA2, p-STAT3, and cleaved caspase3, but not Hsp70, in rat hearts. (a) Representative western blot bands. (b) Hsp70 protein expression. (c) p-p38 MAPK protein expression. (d) SERCA2 protein expression. (e) p-STAT3 protein expression. (f) Cleaved caspase3 protein expression. *n* = 5. Data are shown as means ± SEM. ^∗∗^*P* < 0.01 vs. the sham group; ^#^*P* < 0.05, ^##^*P* < 0.01 vs. the I/R group. I/R: ischemia/reperfusion.

Inhibition of p38 MAPK by SB203580 pretreatment significantly improved myocardial infarct size (Figures 6(a)–6(b)), reduced serum cTnI level (Figure 6(c)), and alleviated [Ca^2+^]_i_ overload (Figure 6(d)) during I/R-induced myocardial injury in rats. Myocardial I/R also significantly elevated the mRNA expression of IL-1*β* (Figure 6(e)) and IL-6 (Figure 6(f)) as well as myocardial apoptosis rate (Figures 6(g)–6(h)), which was effectively inhibited by SB203580. Moreover, the protein expression of Hsp70, p-p38 MAPK, p-STAT3, and cleaved-caspase3 were upregulated and SERCA2 was downregulated during myocardial I/R, while SB203580 pretreatment partly reversed most of these changes (Figure 7). In line with the results in cells, SB203580 did not affect the expression of Hsp70.

### 3.5. Knockdown of Hsp70 Aggravated OGD/R-Induced Injury through p38 MAPK Signaling

**Figure 8:**
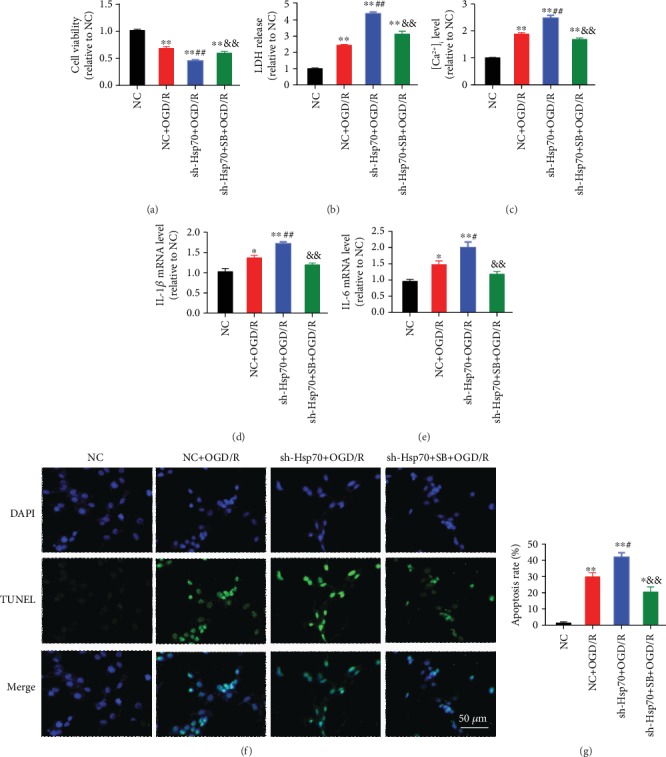
Aggravation of OGD/R-induced cell injury by Hsp70 knockdown was blocked by p38 MAPK inhibition. (a) shRNA-Hsp70 further reduced cell viability during OGD/R, which was abolished by SB203580 (*n* = 8). (b) Increase in LDH release by shRNA-Hsp70 was blocked by SB203580 (*n* = 8). (c) shRNA-Hsp70 further increased [Ca^2+^]_i_ level during OGD/R, which was inhibited by SB203580 (*n* = 5). (d and e) shRNA-Hsp70 further activated IL-1*β* and IL-6 mRNA expression, which was blocked by SB203580 (*n* = 5). (f) Representative TUNEL staining images. Scale bar = 50 *μ*m. (g) shRNA-Hsp70 further increased cell apoptosis during OGD/R, which was reversed by SB203580 (*n* = 5). Data are shown as means ± SEM. ^∗^*P* < 0.05, ^∗∗^*P* < 0.01 vs. the NC group; ^#^*P* < 0.05, ^##^*P* < 0.01 vs. the NC + OGD/R group; ^&&^*P* < 0.01 vs. the sh − Hsp70 + OGD/R group. NC: negative control transfection; OGD/R: oxygen-glucose deprivation/reoxygenation.

**Figure 9:**
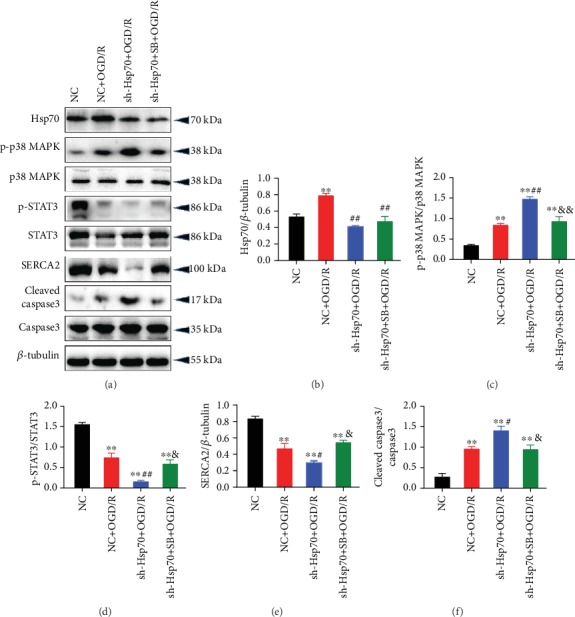
Changes in protein expression of p-p38 MAPK, p-STAT3, SERCA2, and cleaved caspase3 caused by shRNA-Hsp70 in cells were blocked by p38 MAPK inhibition. (a) Representative western blot bands. (b) Hsp70 protein expression. (c) p-p38 MAPK protein expression. (d) p-STAT3 protein expression. (e) SERCA2 protein expression. (f) Cleaved caspase3 protein expression. *n* = 5. Data are shown as means ± SEM. ^∗∗^*P* < 0.01 vs. the NC group; ^#^*P* < 0.05, ^##^*P* < 0.01 vs. the NC + OGD/R group; ^&^*P* < 0.05, ^&&^*P* < 0.01 vs. the sh − Hsp70 + OGD/R group. NC: negative control transfection; OGD/R: oxygen-glucose deprivation/reoxygenation.

Transfection with shRNA-Hsp70 exacerbated OGD/R-induced changes in cell viability (Figure 8(a)), LDH release (Figure 8(b)), and [Ca^2+^]_i_ overload (Figure 8(c)) in neonatal rat cardiomyocytes, while inhibition of p38 MAPK by SB203580 significantly attenuated these effects of Hsp70 knockdown. In addition, the mRNA levels of IL-1*β* (Figure 8(d)) and IL-6 (Figure 8(e)) as well as cell apoptosis rate (Figures 8(f)–8(g)) were further elevated by shRNA-Hsp70 during OGD/R, which was blocked by p38 MAPK inhibition. Regarding the expression of relevant proteins, shRNA-Hsp70 transfection significantly reduced the protein expression of Hsp70 during OGD/R. In addition, knockdown of Hsp70 further increased the protein expression of p-p38 MAPK and cleaved-caspase3 and decreased the expression of SERCA2 and p-STAT3 during OGD/R, while pretreatment with SB203580 reversed these changes induced by shRNA-Hsp70 (Figure 9).

### 3.6. Inhibition of Hsp70 Exacerbated Rat Myocardial I/R-Induced Injury through p38 MAPK Signaling

**Figure 10:**
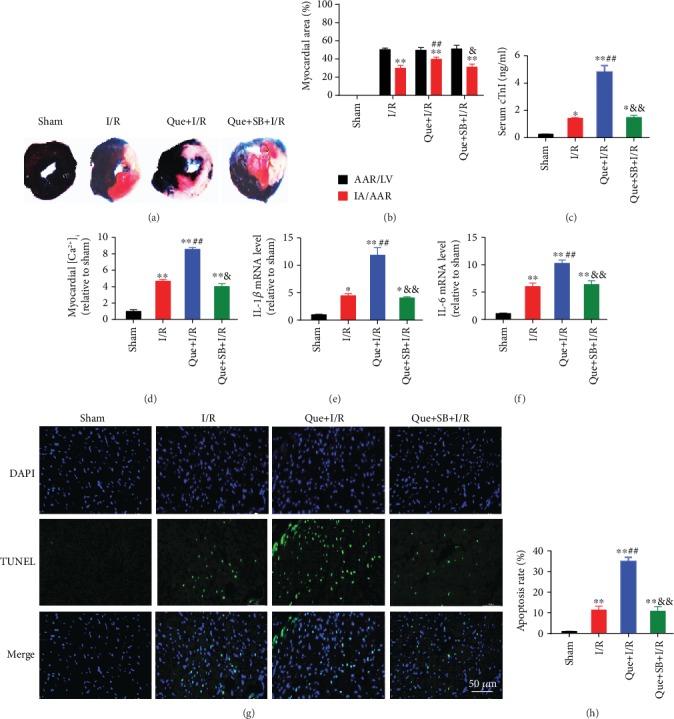
Exacerbation of I/R-induced myocardial injury by Hsp70 inhibition was abolished by p38 MAPK inhibition. (a) Representative Evans blue/TTC staining images. (b) Quercetin, an Hsp70 inhibitor, further increased myocardial infarct size, which was blocked by SB203580. (c) SB203580 blocked quercetin-induced increase in serum cTnI. (d) SB203580 inhibited quercetin-induced increase in [Ca^2+^]_i_ level. (e and f) SB203580 suppressed quercetin-induced IL-1*β* and IL-6 mRNA activation. (g) Representative TUNEL staining images. Scale bar = 50 *μ*m. (h) SB203580 reversed quercetin-induced elevation in cell apoptosis. *n* = 5. Data are shown as means ± SEM. ^∗^*P* < 0.05, ^∗∗^*P* < 0.01 vs. the sham group; ^##^*P* < 0.01 vs. the I/R group; ^&^*P* < 0.05, ^&&^*P* < 0.01 vs. the Que + I/R group. I/R: ischemia/reperfusion; AAR: area at risk; LV: left ventricle; IA: infarct area.

**Figure 11:**
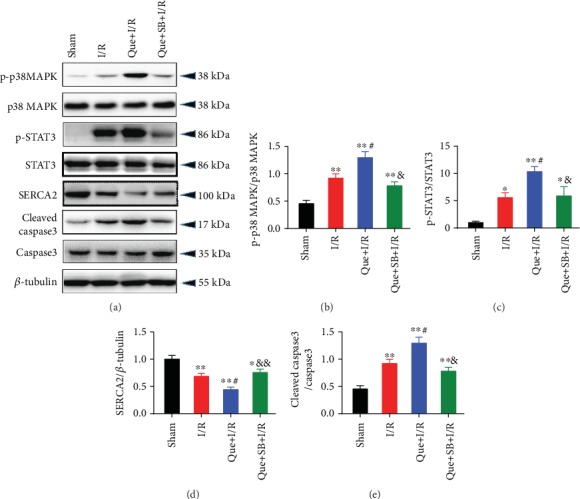
Changes in protein expression of p-p38 MAPK, p-STAT3, SERCA2, and cleaved caspase3 caused by quercetin in myocardial I/R rats were blocked by p38 MAPK inhibition. (a) Representative western blot bands. (b) p-p38 MAPK protein expression. (c) p-STAT3 protein expression. (d) SERCA2 protein expression. (e) cleaved caspase3 protein expression. *n* = 5. Data are shown as means ± SEM. ^∗^*P* < 0.05, ^∗∗^*P* < 0.01 vs. the sham group; ^#^*P* < 0.05 vs. the I/R group; ^&^*P* < 0.05, ^&&^*P* < 0.01 vs. the Que + I/R group. I/R: ischemia/reperfusion.

Inhibition of Hsp70 by quercetin, an Hsp70 inhibitor, significantly increased myocardial infarct size (Figures 10(a) and 10(b)), serum cTnI (Figure 10(c)), and myocardial [Ca^2+^]_i_ level (Figure 10(d)) during I/R-induced myocardial injury in rats. Hsp70 inhibition further activated the expression of IL-1*β* (Figure 10(e)) and IL-6 (Figure 10(f)) and increased cell apoptosis (Figures 10(g) and 10(h)). In terms of the protein expression, Hsp70 inhibition led to upregulation of p-p38 MAPK, p-STAT3, and cleaved-caspase3 and downregulation of SERCA2 during myocardial I/R injury (Figure 11). The above-mentioned effects elicited by quercetin were abolished by inhibiting p38 MAPK with SB203580.

## 4. Discussion

**Figure 12:**
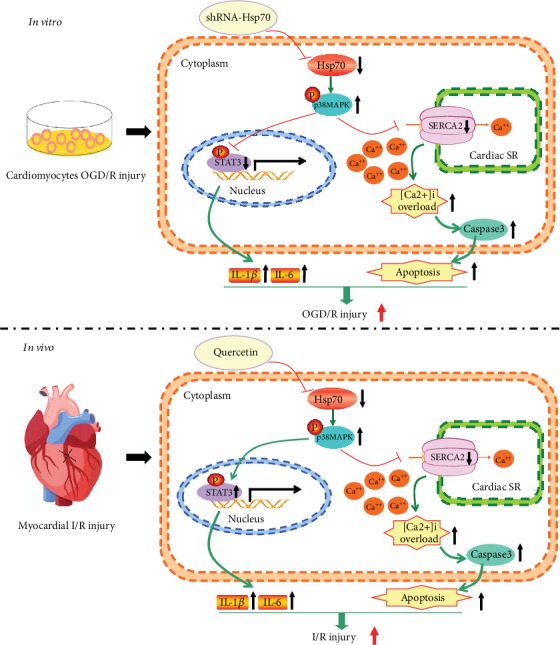
Schematic showing Hsp70 inhibition aggravates cardiac I/R injury through regulating p38 MAPK signaling. *In vitro*, knockdown of Hsp70 increases p38 MAPK phosphorylation, leading to downregulation of SERCA and phosphorylated STAT3 expression. Decreased SERCA activity causes cytosolic Ca^2+^ reuptake dysfunction. Increased cytosolic free Ca^2+^ results in [Ca^2+^]_i_ overload, triggering caspase-3-dependent cell apoptosis. Besides, decreased STAT3 phosphorylation activates the transcription of IL-1*β* and IL-6, promoting inflammatory response. *In vivo*, Hsp70 inhibition enhances the level of p38 MAPK phosphorylation, reduces SERCA activity, and increases STAT3 phosphorylation. Decreased SERCA leads to [Ca^2+^]_i_ overload and cell apoptosis, and increased STAT3 phosphorylation induces inflammatory response by upregulating IL-1*β* and IL-6. OGD/R: oxygen-glucose deprivation/reoxygenation; I/R: ischemia/reperfusion; SR: sarcoplasmic reticulum; [Ca^2+^]_i_: intracellular calcium.

The present study demonstrated that inhibition of Hsp70 exacerbated inflammation, [Ca^2+^]_i_ overload, and apoptosis and modulated the expression of p-p38 MAPK, SERCA2, p-STAT3, and cleaved-caspase3 during OGD/R-induced cell injury and I/R-induced myocardial injury, based on the *in vitro* and *in vivo* experiments. Moreover, inhibition of p38 MAPK phosphorylation significantly attenuated the above-mentioned harmful effects induced by Hsp70 inhibition and attenuated cell death or myocardial injury. These results reveal that cardioprotection offered by Hsp70 was mediated, at least in part, through regulating the p38 MAPK signaling (Figure 12).

Ca^2+^ plays a critical role in the excitation-contraction coupling in cardiac muscle, and Ca^2+^ homeostasis is essential for normal heart function. Previous studies have indicated the effects of Hsp70 on Ca^2+^ homeostasis during cardiac I/R injury. Specifically, blockade of Hsp70 synthesis aggravated Ca^2+^ disorder in rat ventricular myocytes subjected to simulated ischemia [31], while activation of Hsp70 by preconditioning improved the ischemia-impaired Ca^2+^ homeostasis [32]. In addition, p38 MAPK pathway is also involved in the Ca^2+^ regulation. In isolated cardiomyocytes and perfused rat hearts, activation of p38 MAPK signaling induced [Ca^2+^]_i_ overload during the I/R process, while inhibiting p38 MAPK by SB203580 attenuated the I/R-induced injury by enhancing SERCA2*α* activity, reducing [Ca^2+^]_i_ overload, and suppressing apoptosis [24]. As a calcium pump, SERCA2*α* plays a crucial role in the regulation of Ca^2+^ homeostasis by reuptake of cytosolic Ca^2+^ into the sarcoplasmic reticulum [33]. During myocardial I/R injury, decreased SERCA activity leads to contractile dysfunction and triggers calcium-dependent injury [34, 35]. In our study, myocardial I/R induced [Ca^2+^]_i_ overload, upregulation of p-p38 MAPK, and downregulation of SERCA2, which was effectively blocked by inhibition of p38 MAPK with SB203580 pretreatment. Of note, as shown in Figure 8, SB203580 administration led to lower [Ca^2+^]_i_ level and expression of IL-1*β* and IL-6 mRNA compared to the NC + OGD/R group, and the values of IL-1*β* and IL-6 were close to the NC (negative control transfection) group. In addition, SB203580 led to reduced apoptosis rate of ~20% compared to ~30% in the NC + OGD/R group. However, these differences were not statistically significant. As the OGD/R or myocardial I/R is a complex pathophysiological process with a number of underlying mechanisms, targeting only one mechanism is not likely to produce a strong enough protective effect to bring the cells back to the control level. Based on our findings, p38 MAPK is a key factor via which Hsp70 protects against OGD/R-induced cell injury or I/R-induced myocardial injury.

The organ-protective role of Hsp70 has been indicated in several animal models. In focal cerebral ischemia in mice, knockout of Hsp70 significantly increased the infarct volume, suggesting the cerebral protective effect of Hsp70 [36]. In primary rat hypothalamic cells, inhibition of HSP72, a member of the Hsp70 family, aggravated heat-induced cell death, while activation of HSP72 by mild heat preconditioning significantly attenuated cell injury [37]. In addition, Hsp70 takes part in the modulation of immune and inflammatory responses during tissue injury. A previous study showed that macrophages with accumulated Hsp70 had decreased secretion of inflammatory cytokines including tumor necrosis factor-*α* and IL-6 [38]. In our study, inhibition of Hsp70 further enhanced the expression level of IL-1*β* and IL-6 during myocardial I/R injury. However, HSPs can also be released into the extracellular environment to produce a diverse interaction with cells [39]. Extracellular Hsp70 induced cardiomyocyte inflammation and decreased contractility via toll-like receptors and nuclear factor-*κ*B in primary cardiomyocytes [40]. Moreover, long-term overexpression of Hsp70 failed to prevent cardiac dysfunction and adverse remodeling following chronic heart failure and atrial fibrillation [41]. In this context, the role of Hsp70 in cardiac I/R injury still remains unclear. In myocardial I/R mice, Dillmann and colleagues showed that knockout of Hsp70 genes resulted in the development of cardiac hypertrophy after myocardial I/R injury, which may be related to several signaling pathways including Jun N-terminal kinase (JNK), p38 MAPK, Raf-1, and extracellular signal-regulated kinase (ERK) [42]. However, it is still unclear whether Hsp70 modulates p38 MAPK signaling and its effects on [Ca^2+^]_i_ overload and cell apoptosis during myocardial I/R injury. Our study confirmed that the upregulation of Hsp70 played a protective role against OGD/R-induced cell injury in neonatal rat cardiomyocytes and I/R-induced myocardial injury in rats through the inhibition of p38 MAPK signaling.

STAT3 is a transcription factor involved in myocardial I/R injury [43, 44]. Studies showed that the activation of STAT3 reduced myocardial infarct size through suppressing the expression of proapoptotic protein caspase3 [45, 46]. A recent study also indicated that promoting STAT3 phosphorylation alleviated I/R injury in mouse hearts [47]. These results were consistent with the findings in our *in vitro* experiment. We found that p38 MAPK inhibition attenuated OGD/R-induced injury in neonatal rat cardiomyocytes through increasing STAT3 phosphorylation and inhibiting apoptosis and inflammation. However, there is a discrepancy of STAT3 expression between the cell and rat experiments in our study. In rats, we found that p38 MAPK inhibition protected the heart against I/R injury, which was associated with decreased STAT3 phosphorylation. Similarly, Szczepanek et al. [48] showed that transcriptionally inactive STAT3 significantly alleviated myocardial I/R injury and improved survival rate in mice. In other organs, inhibition of STAT3 signaling reduced renal or intestinal I/R injury through the suppression of caspase3-dependent apoptosis [49, 50]. Regarding the effects of STAT3 on inflammation, one study showed that the activation of phosphorylated STAT3 during myocardial I/R reduced the levels of inflammatory factors such as IL-6 and TNF-*α*, but other studies found that suppressing JAK2/STAT3 signaling resulted in lower levels of IL-6, IL-8, and TNF-*α* [51, 52]. Therefore, STAT3 activation and phosphorylation could be beneficial or detrimental based on different conditions. In this context, more studies are still needed to verify the role of STAT3 in I/R-induced cardiac injury.

This study has several limitations. First, this study investigated OGD/R-induced cell injury and I/R-induced myocardial injury in an early stage, i.e., reoxygenation for 2 h and reperfusion for 30 min. The myocardial infarct size was nearly doubled at 24 h compared to 2 h, which was in line with the changes in serum cTnI. The protein expression of Hsp70 and p-p38 MAPK peaked at 30 min, causing significant calcium overload in the early period of reperfusion. These results indicate a progressive cardiac injury during reperfusion, although several indices gradually decreased. It is also possible that other mechanisms were involved in the late period of reperfusion. Second, as this study aimed to explore whether targeting Hsp70 could affect myocardial I/R injury through regulating p38 MAPK signaling, the interaction between p38 MAPK and STAT3 was not evaluated. Third, the current results suggest that the upregulation of Hsp70 was protective against myocardial I/R injury. However, it is still hard to extrapolate that overexpression of Hsp70 could exert further cardioprotection. Last, whether the regulation of p38 MAPK signaling by Hsp70 underlies ischemic preconditioning during myocardial I/R process also needs to be investigated.

In conclusion, this study revealed that inhibition of Hsp70 aggravated myocardial I/R-induced cardiomyocyte inflammation, [Ca^2+^]_i_ overload, and caspase3-dependent apoptosis through regulating the p38 MAPK signaling pathway. These results address the important role of Hsp70 and p38 MAPK during I/R-induced myocardial injury, which may provide novel insight into the therapeutic targets for cardioprotection.

## Figures and Tables

**Table 1:** Arterial blood analysis.

	Sham (*n* = 6)	I/R-30 min (*n* = 6)	I/R-2 h (*n* = 6)	I/R-24 h (*n* = 6)
pH	7.39 ± 0.03	7.39 ± 0.03	7.40 ± 0.02	7.40 ± 0.03
PaCO_2_ (mmHg)	37.67 ± 1.80	37.7 ± 2.20	39.33 ± 2.06	40.17 ± 2.27
PaO_2_ (mmHg)	95.83 ± 2.97	95.33 ± 3.25	96.83 ± 2.79	96.50 ± 2.99
Hct (%)	36.50 ± 2.26	40.33 ± 3.35	41.67 ± 4.96	41.00 ± 3.51
Hb (g/dl)	12.83 ± 1.05	12.75 ± 1.04	13.58 ± 1.65	13.42 ± 1.25
Na^+^ (mmol/L)	142.00 ± 1.53	143.00 ± 1.91	142.17 ± 2.48	142.83 ± 1.57
K^+^ (mmol/L)	4.07 ± 0.20	3.93 ± 0.28	4.28 ± 0.23	4.17 ± 0.34

PaCO_2_: partial pressure of arterial carbon dioxide; PO_2_: partial pressure of arterial oxygen; Hct: hematocrit; Hb: hemoglobin; Na^+^: sodium; K^+^: potassium.

## Data Availability

The data used to support the findings of this study are available from the corresponding authors upon request.
